# Diverse antitumor effects of ascorbic acid on cancer cells and the tumor microenvironment

**DOI:** 10.3389/fonc.2022.981547

**Published:** 2022-09-20

**Authors:** Takeru Maekawa, Toru Miyake, Masaji Tani, Shinji Uemoto

**Affiliations:** ^1^Division of Gastrointestinal, Breast, Pediatric, and General Surgery, Department of Surgery, Shiga University of Medical Science, Otsu, Japan; ^2^Shiga University of Medical Science, Otsu, Japan

**Keywords:** ascorbic acid, antitumor effect, cancer, cancer-associated fibroblast, antioxidant, intravenous administration

## Abstract

Ascorbic acid has attracted substantial attention for its potential antitumor effects by acting as an antioxidant *in vivo* and as a cofactor in diverse enzymatic reactions. However, solid proof of its clinical efficacy against cancer and the mechanism behind its effect have not been established. Moreover, cancer forms cancer-specific microenvironments and interacts with various cells, such as cancer-associated fibroblasts (CAFs), to maintain cancer growth and progression; however, the effect of ascorbic acid on the cancer microenvironment is unclear. This review discusses the effects and mechanisms of ascorbic acid on cancer, including the role of ascorbic acid concentration. In addition, we present future perspectives on the effects of ascorbic acid on cancer cells and the CAF microenvironment. Ascorbic acid has a variety of effects, which contributes to the complexity of these effects. Oral administration of ascorbic acid results in low blood concentrations (<0.2 mM) and acts as a cofactor for antioxidant effects, collagen secretion, and HIFα degradation. In contrast, intravenous treatment achieves large blood concentrations (>1 mM) and has oxidative-promoting actions that exert anticancer effects *via* reactive oxygen species. Therefore, intravenous administration at high concentrations is required to achieve the desired effects on cancer cells during treatment. Partial data on the effect of ascorbic acid on fibroblasts indicate that it may also modulate collagen secretion in CAFs and impart tumor-suppressive effects. Thus, future studies should verify the effect of ascorbic acid on CAFs. The findings of this review can be used to guide further research and clinical trials.

## Introduction

Ascorbic acid, also known as vitamin C, is a low-molecular-weight compound with the chemical formula C_6_H_8_O_6_ and a molecular weight of 176.12 g/mol. It is an essential water-soluble vitamin that cannot be synthesized in the human body (1). Instead, this vitamin must be acquired by consuming food. Inadequate provision of dietary vitamin C can lead to deficiencies such as scurvy (2–4). Ascorbic acid acts *in vivo* as an antioxidant and cofactor in various enzymatic reactions but has also attracted substantial attention for its potential antitumor effects (5, 6). However, the clinical efficacy of ascorbic acid as an anticancer treatment, and the mechanism behind its effects, have not yet been confirmed.

Cancer maintains its characteristic growth and progression by interacting with surrounding cells, forming a cancer microenvironment composed of various cells. Among these cells, cancer-associated fibroblasts (CAFs) play a significant role in cancer cell proliferation, invasion, and metastasis by providing growth factors and nutrients to cancer cells and reorganizing the extracellular matrix of the peri-cancer stroma (7–11). However, the effect of ascorbic acid on the cancer microenvironment is unclear. Moreover, the heterogeneity phenotype of fibroblasts in the peritumoral stroma of some carcinomas promotes tumor growth (12, 13). Therefore, elucidation of the heterogeneity of fibroblasts is urgently required for the effective destruction of cancer cells.

In this review, we discuss the differences between the antioxidant and oxidant-promoting effects of ascorbic acid, including the role of ascorbic acid concentration. Our current understanding of the concentration-dependent actions and processes of ascorbic acid is also explained. We then provide future perspectives on the antitumor effects of ascorbic acid on cancer cells and its effects on CAFs, which form a key cancer microenvironment.

## Administration route and vascular concentration of ascorbic acid

Orally ingested ascorbic acid is absorbed by transporters of sodium-dependent vitamin C transporters (SVCTs) and glucose transporters (GLUTs) in the small intestine and excreted *via* the kidneys (14). *In vivo*, ascorbic acid exists as reduced ascorbic acid or oxidized ascorbic acid (dehydroascorbic acid (DHA)), which are respectively taken into cells through SVCTs and GLUTs (15–17). Rat experiments revealed variations between the oral and intravenous administration of ascorbic acid, whereby oral administration of 5 mg/g of body weight did not raise blood ascorbic acid concentrations, but intravenous administration of 5 mg/g boosted ascorbic acid concentrations to approximately 10 mM (18). However, since mice and rats can synthesize ascorbic acid in their bodies (<100 μM), it is necessary to be careful in applying the results of experiments with mice and rats to humans, whose systems cannot synthesize ascorbic acid. In human studies, oral administration of 400 mg or more of ascorbic acid maintained steady-state blood concentrations of 50–80 µM (19), with oral administration of 3 g of ascorbic acid every 4 h increasing the maximum blood concentration to approximately 220 µM. Conversely, intravenous administration of 50 g of ascorbic acid was predicted to increase the maximum blood concentration to approximately 13.4 mM (20). The half-life of ascorbic acid in the blood is 2.0 ± 0.6 h (21). Furthermore, in a report on patients with cancer, ascorbic acid concentrations in the blood reached 20.3–49.0 mM with intravenous administration of 60–70 g/m^2^ or 1.5 g/kg of ascorbic acid (21–23). In other words, blood concentrations of ascorbic acid vary widely depending on the route of administration. Thus, the pharmacological effects of ascorbic acid resulting from the low concentrations achieved by oral administration (several hundred μM) may differ from those resulting from the high pharmacological concentrations achieved by intravenous administration (>1 mM). As such, the intended administration route of ascorbic acid must be considered. Adverse effects of ascorbic acid include effects on renal function and hemolysis caused by a deficiency of glucose-6-phosphate dehydrogenase (G6PD). Oral doses of more than 1000 mg per day increase renal excretion of urate and oxalate compared to lower doses, so caution should be exercised when administering high doses (19). G6PD is required for the proper function of glutathione peroxidase, especially in erythrocytes (24). However, many clinical trials in which high concentrations of intravenous ascorbic acid were administered as monotherapy or in combination with anticancer agents have shown no serious adverse effects (21, 25–27). Therefore, ascorbic acid is considered a drug with very low toxicity to the human body.

## *In vivo* effects of ascorbic acid

Recent studies have demonstrated that ascorbic acid absorbed *in vivo* has both antioxidant and oxidant-promoting effects (28, 29). Ascorbic acid also exhibits various physiological effects by catalyzing Fe(II)- and 2-oxoglutarate-dependent dioxygenase reactions (14).

### Ascorbic acid and reactive oxygen species

Ascorbic acid degrades reactive oxygen species (ROS) at average blood concentrations of 40–80 μM, reducing low-density lipoprotein oxidation associated with atherosclerosis and lipid oxidation of cell membranes (30–32). However, high pharmacological concentrations of ascorbic acid achieved *via* intravenous administration produce H_2_O_2_ *in vivo* (18, 33, 34) and then hydroxyl radicals *via* the Fenton reaction (35). Intravascularly, ROS produced by high concentrations of ascorbic acid are degraded by catalase in serum, whereas extravascularly, ROS accumulate without degradation by ascorbic acid and act as a pro-oxidant. Thus, ascorbic acid is notable for its paradoxical activity, serving as an antioxidant at low doses and a pro-oxidant at high doses (28, 29). In addition, oral administration of ascorbic acid does not reach the same pharmacological concentrations as intravenous treatment (19, 20); therefore, intravenous administration of ascorbic acid is required for pro-oxidant activity to occur. In a rat study, intravenous administration of 0.5 mg/g of ascorbic acid increased the H_2_O_2_ concentration in the extracellular fluid from undetectable to 20 μM, and intraperitoneal injection of the same dose increased H_2_O_2_ concentration to approximately 5 μM. In contrast, no increase in H_2_O_2_ concentration in the extracellular fluid was detected after oral administration of ascorbic acid (18). In addition, in a mouse subcutaneous transplantation model, intraperitoneal administration of 4 mg/g of ascorbic acid increased the H_2_O_2_ concentration in the extracellular fluid around the tumor to approximately 150 μM (34, 35).

### Ascorbic acid as a cofactor for dioxygenase

Members of the Fe(II) and 2-oxoglutarate-dependent dioxygenase families catalyze many oxidation reactions throughout biology. Ascorbic acid acts as a coenzyme that catalyzes the reactions that produce hydroxylation products using 2-oxoglutarate and oxygen as substrates (36). Particularly well-known are the reactions in collagen (37) and HIFα, which is a master regulator of the cellular hypoxia response pathway (38). The reaction in collagen is mediated by one of the proline hydroxylases, collagen prolyl-4-hydroxylase (C-P4H), which hydroxylates the procollagen proline (37). C-P4H has a high binding capacity to oxygen and is not affected by the oxygen concentration. Conversely, in the reactions in HIFα, ascorbic acid catalyzes two types of reactions: PHD1-3 in proline hydroxylase (38–40) and factor inhibiting HIF-1 (FIH-1) in asparagine hydroxylase (41–43). In the PHD reaction, ascorbic acid degrades HIFα *via* ubiquitination by pVHL proteins (44–46). In the reaction of FIH-1, it suppresses the interaction with CBP/p300, which is a transcriptional cofactor, and suppresses the transcriptional activity of HIFs (42). These reactions are dependent on the oxygen concentration; thus, ascorbic acid acts as an oxygen sensor in the cell because the reaction is reduced in a hypoxic environment and HIF is not degraded (36). The concentrations of ascorbic acid necessary to sustain enzymatic activity of PHDs and FIH-1 are 140-180 uM and 260 uM, respectively (37) and are well above the steady-state blood concentrations of 40–80 μM, suggesting that these reactions require sufficient blood and tissue concentrations of ascorbic acid (14, 47). Ascorbic acid also acts as a cofactor for ten-eleven translocations (TETs) of DNA hydroxylases; TETs are proteins that convert 5-methylcytosine (mC) to 5-hydroxymethylcytosine (hmC) (48, 49). Ascorbic acid promotes DNA demethylation by accelerating the reaction of TETs (50).

## Antitumor effect of ascorbic acid

Ascorbic acid exhibits antitumor effects in various carcinomas (5, 6, 51); however, clinical studies have not yet produced any significant evidence of these effects (52). Ascorbic acid exhibits antitumor effects through ROS-mediated mechanisms and as a cofactor. The mechanisms of ascorbic acid as a cofactor include effects on HIFα *via* PHDs and FIH-1 and epigenetic effects *via* DNA demethylases (6, 49). (**Table 1**) Ascorbic acid can also modulate metabolism and epigenetic gene expression in immune cells as well as cancer cells (64–67). Ascorbic acid is also known to inhibit EMT of tumor cells (58, 59). Here, we discuss the known antitumor effects of ascorbic acid.

**Table 1:** The types and effects of Fe (II) and 2-oxoglutaric acid-dependent dioxygenases in which ascorbic acid acts as a cofactor.

	Collagen prolyl hydroxylases (C-P4H)	Proline hydroxylases	Factor inhibiting HIF-1	DNA/histone demethylases (TETs/JHDMs)
Effect	Promotes collagen production by stabilizing the three-dimensional structure of procollagen through hydroxylation of its proline.	Degrade HIFα *via* ubiquitination by pVHL proteins.	Inhibits the transcriptional abilities of HIF1α *via* the interaction with CBP/p300.	Promote DNA demethylation and regulate epigenetic gene expression.
Ascorbic acid concentration in previous reports	100 μM (53–56)	25-1000 μM (36, 46, 57)		100-2000 μM (58–63)
Antitumor effects	Not clear.	Inhibit tumor cell proliferation by inhibiting angiogenesis and suppressing the promotion of glycolysis.		Reexpresses tumor suppressor genes and suppresses oncogenes. Prevent migration and metastasis by suppressing EMT of tumor cells.

C-P4H, collagen prolyl-4-hydroxylases; TETs, ten-eleven translocation enzymes; JHDMs, Jumonji-domain histone demethylases; HIFα, hypoxia inducible factor α; pVHL protein, the von Hippel-Lindau protein; CBP, CREB-binding protein; EMT, epithelial-mesenchymal transition.

### ROS-mediated antitumor effects of ascorbic acid

The ROS-mediated mechanism is the most well-known mechanism of the antitumor effect of ascorbic acid in various carcinoma. Intravenous administration of high ascorbic acid concentrations acts as a pro-oxidant *in vivo*, producing ROS through the Fenton reaction (18). H_2_O_2_, a ROS formed outside of the cell, diffuses rapidly inside the cell (68) where it consumes antioxidants such as reduced glutathione and NADPH. In addition, in colorectal cancer with KRAS or BRAF mutations, lung cancer with KRAS mutations, and pancreatic cancer, GLUT1 expression is increased because of an accelerated glycolytic pathway, resulting in higher DHA absorption (69–72). ROS accumulation increases oxidative stress, such as DNA damage, and DNA damage increases PARP activity, thereby decreasing NAD^+^ levels and limiting glycolytic system processes (73, 74). In addition, GAPDH, an enzyme of the glycolytic system, is inhibited in its enzymatic function by the reversible binding of oxidized glutathione to cystein152, which is reactive to oxidative stress (75). As a result, the glycolytic pathway produces less adenosine triphosphate (ATP), and cells suffer apoptosis. Indeed, in a report of metabolic changes induced by ascorbate in a colon cancer cell line with KRAS or BRAF mutations, metabolomic analysis using LC/MS/MS showed that upstream metabolites in the glycolytic reaction catalyzed by NAD^+^ and GAPDH were accumulated, whereas downstream metabolites were reduced (74). Because of redox imbalance, cancer cells are susceptible to ROS and the effects of ascorbic acid (76). In conclusion, the pro-oxidant effect of high doses of ascorbic acid induces cell death by generating ROS in cancer cells and limiting ATP generation through the glycolytic pathway.

Conversely, the balance of oxidative stress and antioxidant activity plays a crucial role in tumor development and progression. In melanoma, ROS are overproduced by mitochondria or NADPH oxidase, which promotes tumor development and progression through DNA damage-induced mutation of oncogenes and signal transduction *via* NF-κB (77, 78). In addition, melanoma acquires metastatic potential due to enhanced production of antioxidant enzymes such as catalase and tolerance to oxidative stress (78, 79). Ascorbic acid has a dual impact on melanoma, with high concentrations triggering cell death and low amounts promoting tumor growth (80).

Despite the above reported antitumor effects of ascorbic acid at high concentrations, the ROS-mediated antitumor effects of ascorbic acid remain insufficient for the following reasons. First, the Fenton reaction-mediated ROS-generating effect of ascorbic acid, which is recognized *in vitro*, may be inhibited at *in vivo* concentrations of Fe2^+^ and Fe3^+^ (81). Second, *in vivo*, iron ions are always chelated, so the Fenton reaction may not occur (30). Finally, the inhibitory effect of ascorbic acid on ATP synthesis, even in the presence of PARP inhibitors *in vitro*, may be exerted by ascorbic acid regardless of the reduction of NAD^+^ levels by PARP (82). In conclusion, it is possible that *in vitro* results of the ROS-mediated antitumor effects of ascorbic acid are not compatible with its *in vivo* mode of action, suggesting that alternative anticancer mechanisms may be involved.

### HIFα-mediated antitumor effects of ascorbic acid as a coenzyme

HIFα, which is expressed in many tumors such as melanoma, leukemia, and carcinomas, including colon, pancreatic, and lung cancer (83–87), is involved in angiogenesis and regulation of the glycolytic system, which are crucial processes for cancer growth and progression, suggesting that HIFα may be a novel cancer therapeutic strategy (88, 89). Ascorbic acid is a cofactor for Fe(II)- and 2-oxoglutarate-dependent dioxygenases and has various physiological effects, catalyzing the interaction of PHDs and FIH-1 and degrading the HIFα activity (44–46). Ascorbic acid concentrations in human tumor samples were negatively connected with HIF1α expression in colon cancer, with higher ascorbic acid concentrations associated with prolonged recurrence-free survival (83). In human endometrial tumors, patients with higher ascorbic acid levels in tumors had lower protein expression of HIF1α, VEGF, and GLUT1 and lower malignancy (90). In human pancreatic cancer cell lines, *in vitro*, low ascorbic acid concentrations (25 μM) reduced HIF1α expression and suppressed tumor growth under hypoxic conditions (57). In a model of subcutaneous lung tumor transplantation in rats, intraperitoneal injection of ascorbic acid (1 g/kg) suppressed HIF1α expression in tumors and decreased tumor growth and vascular density (91). In a mouse model of human B cell lymphoma implanted subcutaneously, oral treatment of ascorbic acid (5 g/L) reduced HIF1α expression and prevented tumor development (92).

Thus, the activity of ascorbic acid as a coenzyme may suppress HIFα expression and activity in tumor cells and may inhibit tumor cell proliferation by inhibiting angiogenesis.

### Ascorbic acid regulates epigenomic modifications

Ascorbic acid catalyzes the reaction of DNA hydroxylase TETs and Jumonji C domain-containing histone demethylases (JHMDs), thereby having epigenetic antitumor effects (6, 49, 93). TET is a member of the same family of iron- and 2-oxoglutarate-dependent dioxygenases as PHDs, which convert 5-methylcytosine (5mC) into 5-hydroxymethylcytosine, promoting histone demethylation and contributing to oncogene suppression and the re-expression of tumor suppressor genes (94). In hematologic tumors such as acute myeloid leukemia and myelodysplastic syndromes, loss-of-function mutations in TET2 are known to occur frequently, resulting in decreased and hypermethylated 5hmC. In these hematologic tumors, administration of several hundred μM ascorbic acid has a gene reprogramming effect that restores TET function and increases 5hmC levels, suppressing cell proliferation and promoting myeloid progenitor cell differentiation (60, 94). In malignant melanoma, 5hmC is known to decrease as the disease progresses, and administration of 100 μM ascorbic acid restores 5hmC *via* TET, induces apoptosis in tumor cells, and shows antitumor effects (61, 62). For colon cancer, administration of 1 mM ascorbic acid has also been reported to increase 5hmC *via* TET *in vitro*, showing antitumor effects when combined with an inhibitor of isocitrate dehydrogenase (IDH) mutations (63). JHDMs are histone demethylases that use Fe2 + and α-ketoglutarate as cofactors to demethylate histones and regulate gene expression (95). Isocitrate dehydrogenases (IDH) mutations reduce α-ketoglutarate, a substrate for TETs and JHDMs, and promote DNA methylation in cells with IDH mutations, regulating gene expression that leads to carcinogenesis such as glioma (96). Ascorbic acid is necessary for the proper activity of JHDMs and may correct gene expression that promotes oncogenesis by promoting histone demethylation (93, 97). Essentially, ascorbic acid has antitumor effects by improving the hypermethylation state observed in tumors *via* TETs and JHDMs, and by reprogramming gene expression.

### Ascorbic acid downregulates EMT

Ascorbic acid regulates the epithelial-mesenchymal transition (EMT), which is important for metastatic tumor potential. *In vitro*, ascorbic acid suppressed EMT in human pancreatic cancer cells by decreasing Snail and increasing E-cadherin at concentrations of 1-1.5 mM (98). Ascorbic acid, in conjunction with 5-azacytidine (5-AZA), a potent DNA methyltransferase inhibitor, regulated EMT inhibition and cell cycle progression in human HCC cells *in vitro* by suppressing Snail expression *via* TET (58). Interestingly, ascorbic acid produced two distinct reactions in human breast cancer *in vitro*. A low dose (100 μM) of ascorbic acid decreased E-cadherin and increased the mesenchymal marker vimentin, while a high dose (2 mM) of ascorbic acid conversely increased E-cadherin and decreased vimentin, reversing TGF-β 1-induced EMT and, as a result, suppressing the formation of lung metastases *in vivo* (59). Ascorbic acid at concentrations of 1 mM or higher is thought to suppress EMT in tumors, possibly by inhibiting the effect of TGF-β1 or by regulating Snail expression by TETs.

## Effects of ascorbic acid on fibroblasts

Ascorbic acid is known to enhance collagen synthesis (99, 100) and wound healing (101), reduce UV-induced damage (102, 103), and exhibit anti-inflammatory effects (104, 105); however, these effects are primarily skin-confined. Recently, the CAF cancer microenvironment has attracted considerable attention (10, 11), although few studies have described the effects of ascorbic acid on CAFs. Here, we describe the effects of ascorbic acid on fibroblasts.

### Ascorbic acid and dermal fibroblasts

Ascorbic acid acts as a cofactor for C-P4Hs when taken up by human dermal fibroblasts and promotes collagen production by stabilizing the three-dimensional structure of procollagen through hydroxylation of its proline (4, 106, 107). *In vitro*, a low concentration of ascorbic acid (100 μM) in human skin fibroblasts increases the expression of type1,3,4 collagen and SVCT2 at the mRNA level (53–55) as well as increasing proliferation (56), suggesting a direct effect on fibroblasts. In human clinical data, oral ascorbic acid intake with exercise stimulation doubled the amino-terminal propeptide of collagen I in the blood, indicating enhanced collagen production (108). In addition, ascorbic acid concentrations as low as 0.17 mM in human skin fibroblasts increase the contractile phenotype of myofibroblasts in the presence of TGF-β1 through enhancement of the expression of TGFb1-responsive genes, but do not increase such a phenotype in the absence of TGF-β1 (109). Ascorbic acid promotes collagen production and proliferation of skin fibroblasts as a coenzyme. Moreover, in these studies, ascorbic acid increases in collagen synthesis and secretion occurred at concentrations as low as several hundred μM.

### Ascorbic acid and other fibroblast reports

**Figure 1:**
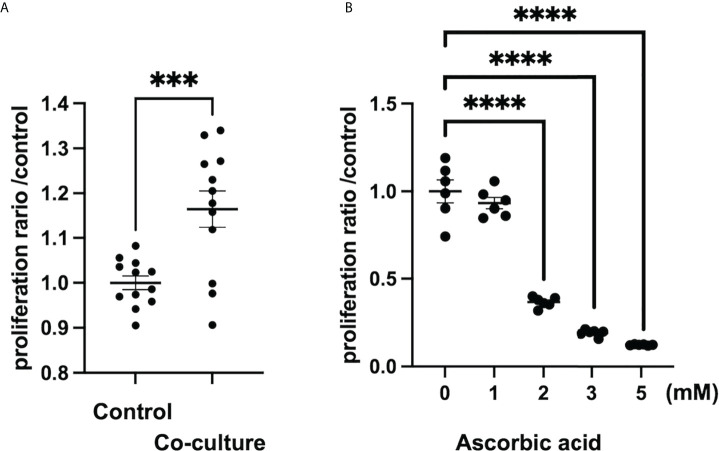
Ascorbic acid reduces the proliferation of human-derived pancreatic fibroblasts (hPFs). **(A)** Proliferation of hPFs is increased in a co-culture with a pancreatic cancer cell line (MIAPaCa2). ***P < 0.001 versus control, means ± SEM, n = 12. **(B)** Proliferation of hPFs is dose-dependently reduced by high-dose ascorbic acid treatment. ****P < 0.0001 versus ascorbic acid 0 mM, means ± SEM, n = 6. Statistical analysis was performed by GraphPad Prism 9 and significance was determined by Student’s t-test.

According to a study on tumor stroma, intraperitoneal administration of ascorbic acid at a high dose of 4 g/kg in an orthotopically transplanted mouse model of human pancreatic cancer resulted in tumor reduction, reduced metastasis, and enhanced tumor stroma due to increased collagen secretion (98). In the 4T1 breast cancer orthotopic model utilizing ascorbic acid-deficient (gulonolactone oxidase knockout mouse) mice, oral administration of ascorbic acid increased type 1 collagen to form a capsule around the tumor, and tumor boundaries were more clearly defined than in the control group (110). Thus, ascorbic acid may increase collagen production in the tumor stroma at both high and low doses. However, it is unknown whether this effect is on tumor cells or CAFs, and further research is needed to determine whether ascorbic acid activates CAFs in the tumor microenvironment and increases collagen production. In contrast, hepatic stellate cells, which are responsible for liver fibrosis, were inhibited *in vitro* by low doses of ascorbic acid (50-200 M), which decreased intracellular TGF-1 in rat cell lines (111). In a report examining the development of pulmonary fibrosis by paraquat treatment, intraperitoneal administration of 150 mg/kg of ascorbic acid inhibited pulmonary fibrosis in a mouse model by inhibiting inflammatory cell infiltration into the bronchoalveolar lavage fluid, suppressing apoptosis by increasing antioxidant activity in the lung, and inhibiting TGF-β in the lung (112). As a result, ascorbic acid may inhibit fibrosis by inhibiting inflammatory cell infiltration and reduction of TGF-β in tissues. In our study, we also found that *in vitro*, human pancreatic-derived fibroblasts, whose proliferation is promoted when co-cultured with cancer cells, receive high doses (>1 mM) of ascorbic acid for growth inhibition. (**Figure 1**) In conclusion, the effects of low and high doses of ascorbic acid on CAFs, such as enhanced collagen production and inhibition of fibrosis development, differ from organ to organ or disease model to disease model and remain unclear.

## Clinical trials on ascorbic acid

In the 1970s, clinical trials involving ascorbic acid revealed that a small sample of patients treated with intravenous and oral ascorbic acid lived longer than a control group (113, 114). At that time, the mechanism of the antitumor effect of ascorbic acid efficacy remained unclear, and subsequent randomized, double-blind, placebo-controlled trials with oral ascorbic acid failed to demonstrate a survival benefit (115, 116). Therefore, the antitumor effect of ascorbic acid was viewed unfavorably. Multiple mechanisms of ascorbic acid’s antitumor effect were subsequently proven *in vitro*, along with differences in ascorbic acid blood levels between oral and intravenous administration methods. Furthermore, the fact that blood levels of ascorbic acid were decreased in cancer patients (117, 118) and that the adverse effects associated with ascorbic acid administration were extremely low, led to the expectation that ascorbic acid could be used for therapeutic applications. (**Table 2**) There were a few scattered case reports showing tumor shrinkage with ascorbic acid treatment (131, 153–158), and there were also reports of antitumor effects in a small number of studies (25, 124, 159, 160). Ascorbic acid in combination with chemotherapeutic agents has also been researched, and some reports of reduced side effects and improved quality of life have been observed (21, 161, 162). In contrast, there have been no large-scale clinical trials that have demonstrated an additional antitumor effect by ascorbic acid (6, 76, 131, 163), and several ongoing clinical trials of ascorbic acid alone or in combination with chemotherapeutic agents for advanced colon cancer, pancreatic cancer, lung cancer, and other malignancies are expected to provide results in the near future (140, 148, 149, 151) (**Table 2**).

**Table 2:** Clinical trials on ClinicalTrials. gov.

Study^a^	Cancer types	Phase of study	Design of study	Therapy	Number of patients (with/without ascorbic acid) or estimated enrollment	Result or primary outcome measures (if trials are not reported)
Published clinical trials						
NCT00954525 (25, 119)	pancreatic cancer	Phase I	single group assignment	Ascorbic acid (IV 50-100 g, 3 infusions per week), gemcitabine and erlotinib for 8 weeks per cycle	9	Seven patients were SD and 2 patients were PD. Time to progression was 89 days (standard deviation 77 days) and overall survival was 182 days (standard deviation 155 days)
NCT00006021 (120, 121)	multiple myeloma	Phase I/II	single group assignment	Ascorbic acid (IV 1 g, 5 infusions per week) and arsenic trioxide for 5 weeks per 7 week	6	Two patients were PR, and 4 patients were SD.
NCT00317811 (122, 123)	multiple myeloma	Phase II	single group assignment	Ascorbic acid (oral 1g, days 1-4 every 2 weeks), bortezomib and melphalan	31	Five patients were CR, 3 patients were VCPR, 6 patients were PR, 9 patients were MR, 6 patients were SD, and 2 patients were PD.
NCT01049880 (124, 125)	pancreatic cancer	Phase I	single group assignment	Ascorbic acid (IV 50-125 g, 2 infusions per week) and gemcitabine	9	Time to progression and overall survival were 26 ± 7 weeks and 13 ± 2 months. (Means ± SEM)
NCT01050621 (26, 126)	all cancer	Phase I/II	single group assignment	Ascorbic acid (IV 1.5 g/kg, 2 or 3 infusions per week) and chemotherapy	14	Three patients had unusually favorable experiences that were deemed highly unlikely to result from chemotherapy alone.
NCT01080352 (127, 128)	prostate cancer	Phase II	single group assignment	Ascorbic acid (IV week 1, 5 g; week 2, 30 g; and weeks 3–12, 60 g, once a week)	23	This treatment was not found to be effective.
NCT01364805 (98, 129)	pancreatic cancer	Phase I/IIa	single group assignment	Ascorbic acid (IV 75-100 g, 3 infusions per week) and gemcitabine	14	Median progression-free survival and median overall survival were 3 months and 15.1 months.
NCT00228319 (130, 131)	ovarian cancer	Phase I/IIa	parallel assignment, randomized	Arm 1: carboplatin and paclitaxel chemotherapy and ascorbic acid (IV 75-100 g, 2 infusion per week) for 6 months/Arm 2: carboplatin and paclitaxel chemotherapy	25 (13/12)	There were no statistically significant difference in overall survival and the median time for disease progression/relapse.
NCT02655913 (132, 133)	non-small-cell lung cancer	Phase I/II	parallel assignment, randomized	Arm 1: administration of ascorbic acid (IV 1 g/kg, 3 infusions per week) for total 25 times, modulated electrohyperthermia, and supportive care/Arm 2: supportive care	97 (49/48)	Progression-free survival (3 months *vs.* 1.85 months, P < 0.05) and overall survival (9.4 months *vs.* 5.6 months, P < 0.05) were significantly prolonged by combination therapy compared to BSC alone.
NCT01905150 (134, 135)	pancreatic cancer	Phase II	parallel assignment, randomized	Arm 1: G-FLIP/G-FLIP-MD and ascorbic acid (IV 75-100 g, 2 infusions per week)/Arm 2: G-FLIP/G-FLIP-MD	26 (we could confirm only abstract, and it did not describe details)	Ascorbic acid may avoid standard 20-40% rates of severe toxicities.
Ongoing or unpublished clinical trials						
NCT01754987 (136)	hepatocellular carcinoma	Phase I/II	parallel assignment, non-randomized	Arm 1: ascorbic acid (IV 100 g, 3 infusions per week) for 16 weeks and sorafenib/Arm 2: sorafenib only	5 (5/0)	Number of participants that experience serious adverse events. (Time Frame: 16 weeks +/- 2 weeks)
NCT03410030 (137)	pancreatic cancer	Phase Ib/II	single group assignment	Ascorbic acid (IV ≥20 mM), nab-paclitaxel, cisplatin, and gemcitabine	36	Disease control rate (CR+PR+SD x18 weeks) (Time Frame: approximately 63 days)
NCT03964688 (138)	multiple myeloma and lymphoma	Phase II	parallel assignment, randomized	Arm 1: ascorbic acid (IV during hospitalization, after oral, total 6 weeks.)/Arm 2: placebo	47	Immune recovery (Time Frame: day 14-28)
NCT02905578 (139)	pancreatic cancer	Phase II	parallel assignment, randomized	Arm 1: ascorbic acid (IV 75 g, 3 infusions per week), gemcitabine, and nab-paclitaxel/Arm 2: gemcitabine and nab-paclitaxel	65	Overall survival (Time Frame: Every 2 months for up to 20 years post-treatment)
NCT03146962 (140)	colorectal, lung, and pancreatic cancer	Phase II	single group assignment	Cohort A: ascorbic acid (IV 1.25 g/kg, 4 infusions per week) for 2-4 consecutive weeks/Cohort B: ascorbic acid (IV 1.25 g/kg, 4 infusions per week) up to 6 months/Cohort C: ascorbic acid (IV 1.25 g/kg, 4 infusions per week) for 1-3 weeks and Yttrium-90 radioembolization of hepatic metastases	78	Change in antitumor activity measured by pathologic response based on tumor regression grading in cohort A patients. (Time Frame: cohort A - 8 weeks) Three-month disease control rate will be evaluated using RECIST v 1.1 in cohort B patients. (Time Frame: Cohort B - 3 months) Maximal tolerated dose of high dose vitamin C in combination with Y90 radioembolization (Time Frame: Cohort C - 16 weeks)
NCT03418038 (141)	high grade B-cell lymphoma with MYC and BCL2 or BCL6 rearrangements, recurrent diffuse large B-cell lymphoma, recurrent Hodgkin lymphoma, recurrent lymphoma, refractory diffuse large B-cell lymphoma, and refractory lymphoma	Phase II	parallel assignment, randomized	Arm A: ascorbic acid (IV) on days 1, 3, 5, 8, 10, 12, 15, 17, and 19, and combination chemotherapy./Arm B: placebo (normal saline) (IV) on days 1, 3, 5, 8, 10, 12, 15, 17, and 19, and combination chemotherapy./Arm C: ascorbic acid (IV) on days 1, 3, 5, 8, 10, 12, 15, 17, and 19, and another combination chemotherapy from Arm A and B.	147	Overall response rate (Arms A and B) (Time Frame: Up to 2 years) Overall response rate (Arm C) (Time Frame: Up to 2 years)
NCT03433781 (142)	myelodysplastic syndromes	Phase Ib/IIa	single group assignment	Ascorbic acid (continuous intravenous infusion/24 hours 50 g, 5 infusions every 4 week)	18	Measure of serum bioavailability of ascorbic acid in Myelodysplastic syndrome patients with ten-eleven translocation 2 mutations (Time Frame: 6 Months)
NCT03508726 (143)	soft tissue sarcoma	Phase Ib/II	single group assignment	Ascorbic acid (IV 62.5 or 75 g, 3 infusions per week)	25	Tumor response as assessed by pCR rate (Time Frame: Start of treatment up to 6 weeks after the last ascorbate infusion)
NCT03682029 (144)	myelodysplastic syndromes, chronic myelomonocytic leukemia-1, and cytopenia	–	parallel assignment, randomized	Arm 1: ascorbic acid (oral 1000 mg, daily) for 12 months/Arm 2: placebo	100	Median change from baseline in variant allele frequency at 12 months (Time Frame: At baseline and at 12 months)
NCT03799094 (145)	non-small-cell lung cancer	Phase I/II	parallel assignment, randomized	Arm 1: ascorbic acid (IV 30 g, once a week) and daily taking tyrosine kinase inhibitor/Arm 2: daily taking tyrosine kinase inhibitor	150	Progression free survival (Time Frame: From the start date of treatment until the date of first documented progression or death, assessed up to 2 years)
NCT03999723 (146)	myelodysplastic syndromes, acute myeloid leukemia, and chronic myelomonocytic leukemia	Phase II	parallel assignment, randomized	Arm 1: ascorbic acid (oral 1000 mg, daily) and azathioprine/Arm 2: placebo and azathioprine	196	Event-free survival (Time Frame: 0-54 months)
NCT04033107 (147)	hepatocellular cancer, pancreatic cancer, gastric cancer, and colorectal cancer	Phase II	single group assignment	Ascorbic acid (IV 1.5 g/kg, D1-3, every 2 weeks) and metformin	30	Progression-free survival (Time Frame: up to 12 weeks)
NCT04046094 (148)	bladder cancer	Phase I/II	single group assignment	Ascorbic acid (IV 25 g, 2 infusions per week) for 4 weeks	21	Post treatment pathological staging (Time Frame: 10 weeks)
NCT04516681 (149)	colorectal cancer	Phase III	parallel assignment, randomized	Arm 1: ascorbic acid (IV 1.5g/kg/day, D1-3, every 2 weeks) and FOLFOXIRI+/- bevacizumab/Arm 2: FOLFOXIRI+/- bevacizumab	400	Objective response rate (Time Frame: up to 5 years)
NCT04634227 (150)	sarcoma, soft tissue sarcoma, unresectable soft tissue sarcoma, metastatic bone tumor, and bone sarcoma	Early Phase I	single group assignment	Ascorbic acid (IV 20-30 mM) on days 1, 2, 8, 9, 15 and 16 of a 28-day cycle, and gemcitabine	20	Determine the 12 weeks progression free survival at 12 weeks post treatment initiation (Time Frame: 12 weeks post-treatment)
NCT04801511 (151)	rectal cancer	Phase II	single group assignment	Ascorbic acid (IV 24 g, 25 times) with preoperative concurrent intensity-modulated radiation therapy and mFOLFOX6 chemotherapy, and then preoperative mFOLFOX6 chemotherapy	60	pCR rate (Time Frame: 2 year From the first subject underwent surgery to the last subject underwent surgery.)
NCT02516670 (152)	prostate cancer	Phase II	parallel assignment, randomized	Arm 1: ascorbic acid (IV 25 g, 2 infusions per week) for 3 weeks and docetaxel/Arm 2: placebo and docetaxel	50	Terminated (insufficient clinical response per DSMB)

a This table describes clinical trials since 2000.

IV, intravenous injection; CR, complete response; VGPR, very good partial response; PR, partial response; SD, stable disease; PD, progressive; pCR, pathologic complete response; DSMB, Data and Safety Monitoring Board.

## Discussion

**Figure 2:**
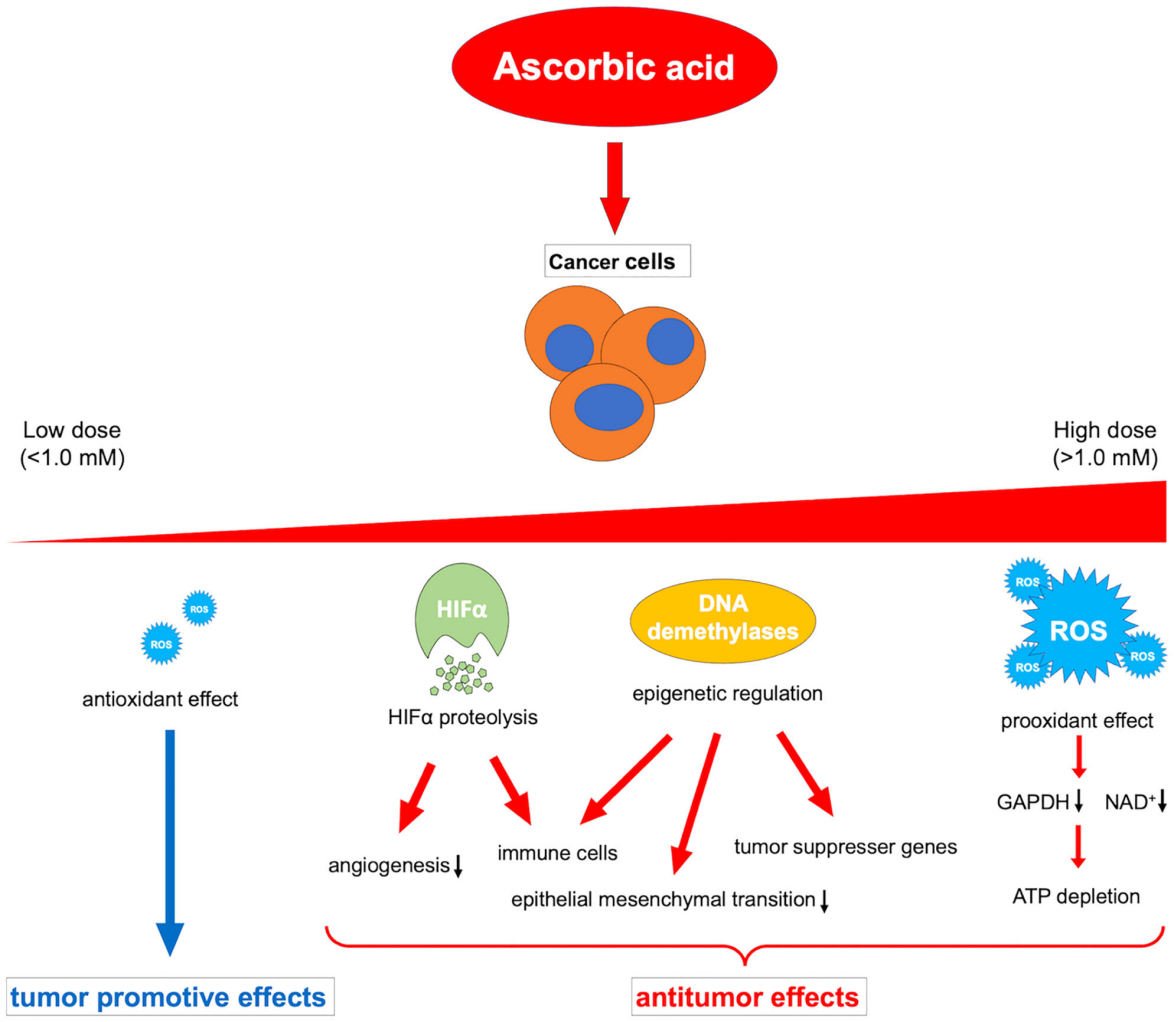
Overview of the various dose-dependent effects of ascorbic acid on cancer. ROS, reactive oxygen species; HIFα, hypoxia-inducible factor-alpha; GAPDH, glyceraldehyde 3-phosphate dehydrogenase; NAD^+^, nicotinamide adenine dinucleotide.

Ascorbic acid is a medicine that has been widely investigated and used for a long time; however, its beneficial effects against cancer have not yet been proven by clinical trials. The contrasting *in vivo* effects of ascorbic acid may explain this. That is, the oxidative-promoting impact at high concentrations is detrimental to cancer cells, whereas the antioxidant effect at low concentrations may promote cancer (164). Because of this paradoxical effect, the administration route of ascorbic acid should be carefully considered. In addition, future research should explain the different activities of multiple dioxylases as cofactors, such as HIFα degradation, immune cell modulation, and epigenetic regulation of gene expression, in relation to the cancer microenvironment (**Figure 2**).

Additionally, research on the effects of ascorbic acid on CAFs implies the existence of novel therapeutic possibilities. Since the diversity of gene expression in fibroblasts *in vivo* differs among organs and pathological conditions (165), the effects of ascorbic acid on CAFs are also expected to vary among organs and pathological conditions. One of the potential effects of ascorbic acid may be the inhibition of tumor-promoting CAFs. Tumor-promoting CAFs support cancer growth by supplying cancer cells with nutrients and growth factors (7–11). Moreover, tumor-promoting CAFs control ECM secretion and protease secretion, remodel the ECM, and generate invasive routes necessary for solid tumor invasion (166, 167). Furthermore, in tumors with a high stromal component, such as pancreatic and breast cancer, the stromal fluid pressure in the tumor area increases, reducing drug delivery and indicating resistance to treatment (168, 169). Tumor-promoting CAFs promote cancer through cross-talk functions with cancer cells, ECM remodeling functions, and physical drug barrier functions. Ascorbic acid has an inhibitory effect on fibroblasts through a reduction in TGF at low doses and an inhibitory effect on cell proliferation *via* a prooxidant effect at higher doses, suggesting that it may have an inhibitory effect on tumor-promoting CAFs.

Conversely, collagen is known to form a barrier that physically obstructs cell migration without protease degradation (170, 171). In a mouse model lacking -SMA-positive fibroblasts, the tumor suppressive effects of CAFs have been demonstrated to induce an undifferentiated tumor phenotype and dramatically reduce survival (172). The increase in cancer stroma, tumor shrinkage, and metastasis inhibition effects of ascorbic acid may be attributed to the activation of tumor suppressive fibroblasts and the formation of collagen barriers that inhibit tumor progression.

**Figure 3:**
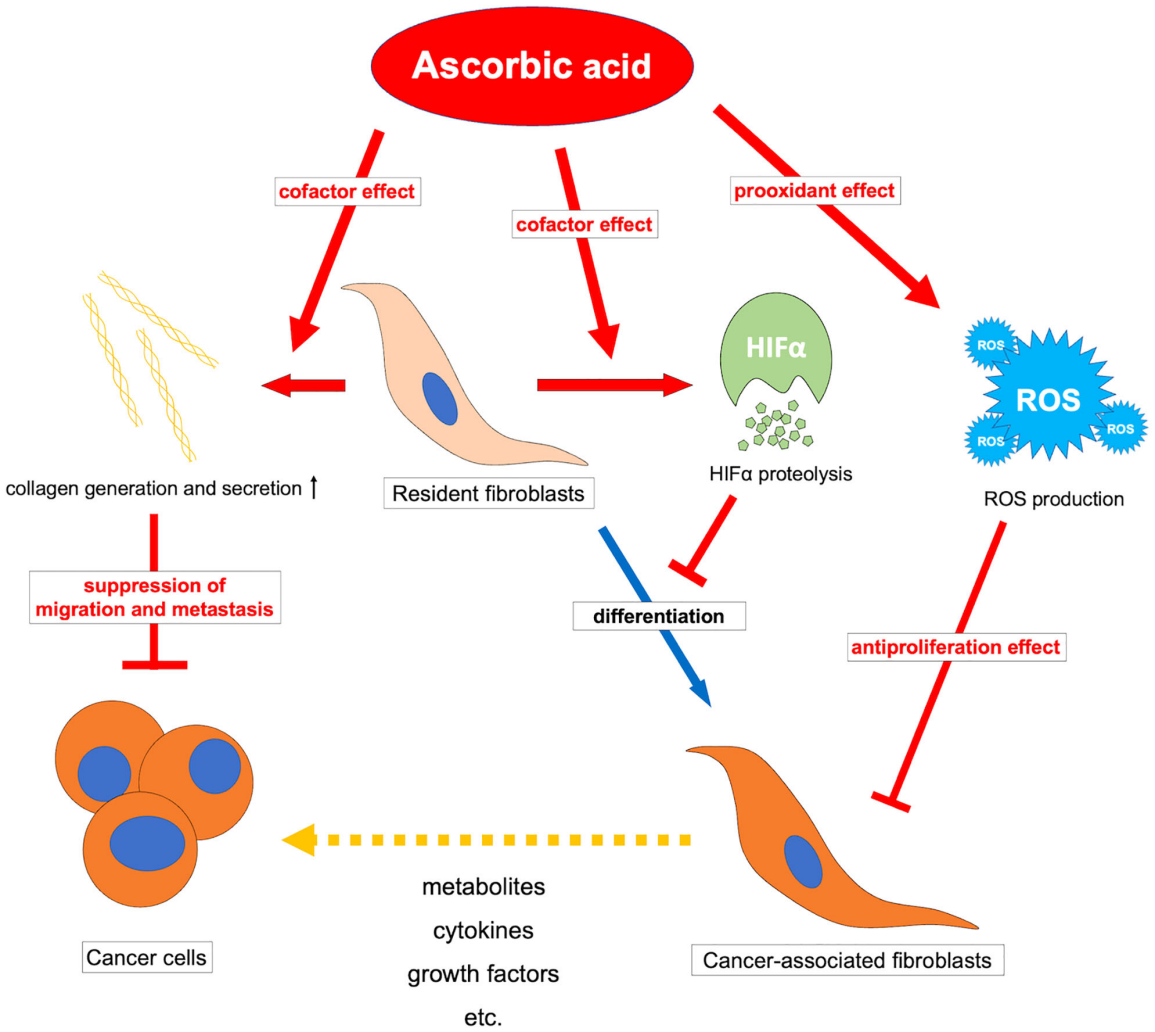
Overview of the antitumor effects of ascorbic acid on cancer-associated fibroblasts. ROS, reactive oxygen species; HIFα, hypoxia-inducible factor-alpha.

Ascorbic acid may also affect CAFs *via* suppression of HIF1α. Tumor-induced ROS-mediated “pseudo-hypoxia” in CAFs leads to the accumulation of HIF1α and enhanced aerobic glycolysis (173, 174). Furthermore, high expression of HIF1α in CAFs induces protein expression in myofibroblasts in CAFs, and inhibition or knockout of HIF1α improves their phenotype (175). Stimulation by TGF-β or PDGF also suppresses IDH3 expression and decreases 2-oxoglutarate in fibroblasts, resulting in HIF1α accumulation and regulating fibroblast differentiation into CAFs (176). Ascorbic acid may inhibit the accumulation of HIF1α by promoting the reaction of 2-oxoglutarate-dependent dioxygenases such as PHDs and FIH-1, thereby suppressing fibroblast differentiation into CAFs. The JAK1/STAT3 pathway is also an important pathway that maintains actomyosin contractility and the CAF phenotype (177), and methylation of the promoter of protein tyrosine phosphatase non-receptor type 6 (SHP-1), which negatively regulates the JAK/STAT pathway, allowing for sustained signaling (167). For this epigenetic reorganization, DNA demethylase-mediated effects such as TETs of ascorbic acid may be exerted. However, CAFs have an enhanced glycolytic system due to chronic hypoxia in the tumor microenvironment and subsequent epigenetic reorganization by demethylation of HIF1α and promoters of enzymes of the glycolytic system (178), and there may be unexpected epigenetic effects of ascorbic acid that should be clarified in the future. Ascorbic acid may have a tumor suppressive effect by affecting CAFs and reprogramming them into normal fibroblasts. (**Figure 3**) It is possible that the antitumor effect of ascorbic acid can be improved by examining the method of administration and adapting it to the expression status of HIF in tumors and CAFs. Furthermore, elucidating the effects of ascorbic acid targeting not only tumor cells but also tumor microenvironments such as CAFs may help to reveal further antitumor effects of ascorbic acid.

## Author contributions

TMa and TMi drafted the manuscript, which was subsequently critically revised by MT and SU. All authors contributed to the article and approved the submitted version.

## Funding

This work was supported by JSPS KAKENHI (grant Nos. 22K08769).

## Acknowledgments

The authors would like to thank Ikuko Arikawa, Yumiko Ito, and Sachiko Sawada for technical assistance with the experiments. Furthermore, we would like to thank Editage (www.editage.jp) for English language editing.

## Conflict of interest

The authors declare that the research was conducted in the absence of any commercial or financial relationships that could be construed as a potential conflict of interest.

## Publisher’s note

All claims expressed in this article are solely those of the authors and do not necessarily represent those of their affiliated organizations, or those of the publisher, the editors and the reviewers. Any product that may be evaluated in this article, or claim that may be made by its manufacturer, is not guaranteed or endorsed by the publisher.
